# *Lactobacillus acidophilus*—Rutin Interplay Investigated by Proteomics

**DOI:** 10.1371/journal.pone.0142376

**Published:** 2015-11-06

**Authors:** Maria Fiorella Mazzeo, Rosa Lippolis, Alida Sorrentino, Sarah Liberti, Federica Fragnito, Rosa Anna Siciliano

**Affiliations:** 1 Institute of Food Sciences, National Research Council, Avellino, Italy; 2 Institute of Biomembranes and Bioenergetics, National Research Council, Bari, Italy; 3 Department of Chemical Sciences, University of Naples Federico II, Naples, Italy; Instituto Nacional de Salud Pública, MEXICO

## Abstract

Dietary polyphenols are bioactive molecules that beneficially affect human health, due to their anti-oxidant, anti-inflammatory, cardio-protective and chemopreventive properties. They are absorbed in a very low percentage in the small intestine and reach intact the colon, where they are metabolized by the gut microbiota. Although it is well documented a key role of microbial metabolism in the absorption of polyphenols and modulation of their biological activity, molecular mechanisms at the basis of the bacteria-polyphenols interplay are still poorly understood. In this context, differential proteomics was applied to reveal adaptive response mechanisms that enabled a potential probiotic *Lactobacillus acidophilus* strain to survive in the presence of the dietary polyphenol rutin. The response to rutin mainly modulated the expression level of proteins involved in general stress response mechanisms and, in particular, induced the activation of protein quality control systems, and affected carbohydrate and amino acid metabolism, protein synthesis and cell wall integrity. Moreover, rutin triggered the expression of proteins involved in oxidation-reduction processes.This study provides a first general view of the impact of dietary polyphenols on metabolic and biological processes of *L*. *acidophilus*.

## Introduction

Dietary polyphenols are bioactive molecules that exert beneficial effects on human health, due to their anti-oxidant, anti-inflammatory, cardio-protective and chemopreventive properties [1,2]. These compounds are secondary plant metabolites found in different vegetables, fruits and beverages such as tea and wine [3]. They are usually classified in flavonoids (whose structures contain two benzene rings linked through a heterocyclic pyrone ring) and nonflavonoid phenolics, a heterogeneous group comprising benzoic acids, stilbenes, lignans, hydrolyzable tannins, gallotannins and ellagitannins. Moreover, polyphenols may be conjugated in nature with various carbohydrates and organic acids. Among flavonols, a class of flavonoids, quercetin is one of the most abundant found in several food matrices, and extensively studied for its relevant biological properties [4]. Rutin (quercetin-3-O-rutinoside) is one of the main glycoconjugated forms of quercetin present in *planta*, and its antioxidant properties and pharmacological benefits for the treatment of chronic diseases such as cancer, diabetes, hypertension and hypercholesterolemia have been recently reviewed [5].

During digestion, polyphenols are absorbed in a very low percentage in the small intestine and reach intact the colon, where they are metabolized by gut microbiota. The transformation of polyphenols occurs via a complex set of chemical reactions (deglycosylation, demethylation, dehydroxylation, decarboxylation and reduction) and leads to the production of a relatively limited number of metabolites, mainly small phenolic acids, regardless of the complexity of natural polyphenols [6–8].

However, microbial metabolism is not only required for polyphenol absorption but also modulates their biological activity. Phenolic compounds produced by the microbial degradation could exhibit an enhanced bioavailability or biological activity compared to those of the native compounds and, in turn, these metabolites could modulate microbiota composition via their antimicrobial activity or pathogen inhibition. Notwithstanding the well documented impact of polyphenols on human health, molecular mechanisms at the basis of the bacteria-polyphenols interaction are still poorly understood and few intestinal bacterial species involved in their metabolism have been identified so far [8,9]. Bifidobacteria and lactobacilli, components of gut microbiota, seemed to be involved in polyphenol degradation as they produced different glycosyl-hydrolases able to efficiently hydrolyze glycoconjugated polyphenols [10]. In particular, the rhamnosidase from *Lactobacillus acidophilus* was able to efficiently degrade flavanone and flavonol glycosides such as hesperidin, naringin and rutin [11]. Therefore, as lactobacilli might contribute to bioavailability and absorption of these compounds, it is crucial to gain insight into the potential interaction between these microorganisms and polyphenols.

A few proteomic studies were carried out to address the effects of polyphenols on lactobacilli metabolism and investigate the molecular mechanisms adopted by these bacteria to grow in the presence of polyphenols. Response mechanisms to tannic acid have been examined in *L*. *hilgardii* and *L*. *plantarum* [12–14]. Rivas-Sendra and coworkers highlighted cytoplasmic proteins presumably involved in the response of *L*. *casei* BL23 to *p*-coumaric acid [15].

As to *L*. *acidophilus*, proteomic studies were performed to assess how environmental stresses affected microbial metabolism [16,17], and to obtain a quantitative profile of bacteriocins present in probiotic preparations [18]. Regarding the probiotic properties of these bacteria, proteins involved in adhesion to intestinal epithelial cells [19,20] and in immunomodulation [21] or associated with the ability of *L*. *acidophilus* to reduce serum cholesterol [22] have been identified. Furthermore, changes in protein expression induced by the prebiotic lactitol have been reported [23]. On the other hand, no proteomic investigation has been carried out on *L*. *acidophilus*-polyphenols interplay. However, it has been documented that a specific intestinal strain of *L*. *acidophilus* showed exceptional resistance to different tea polyphenol extracts, that were able to slow down the growth of other bacteria [24]. Similarly, the polyphenolic extract of *Sesbania grandiflora* flower, containing rutin as a major flavonoid, exhibited a growth promoting property against *L*. *acidophilus* [25]. Furthermore, rutin had no inhibitory effect on representative gut bacteria species and induced a slight growth stimulation of lactobacilli while its aglycone quercetin exhibited a strong growth inhibitory effect [26]. In this context, we carried out the first differential proteomic study on a model system constituted by a potential intestinal probiotic *L*. *acidophilus* strain and rutin. These preliminary results could be considered an initial step to reveal the impact of rutin on *L*. *acidophilus* metabolism and functional processes.

## Materials and Methods

### Bacterial growth and proteome extraction

**Fig 1:**
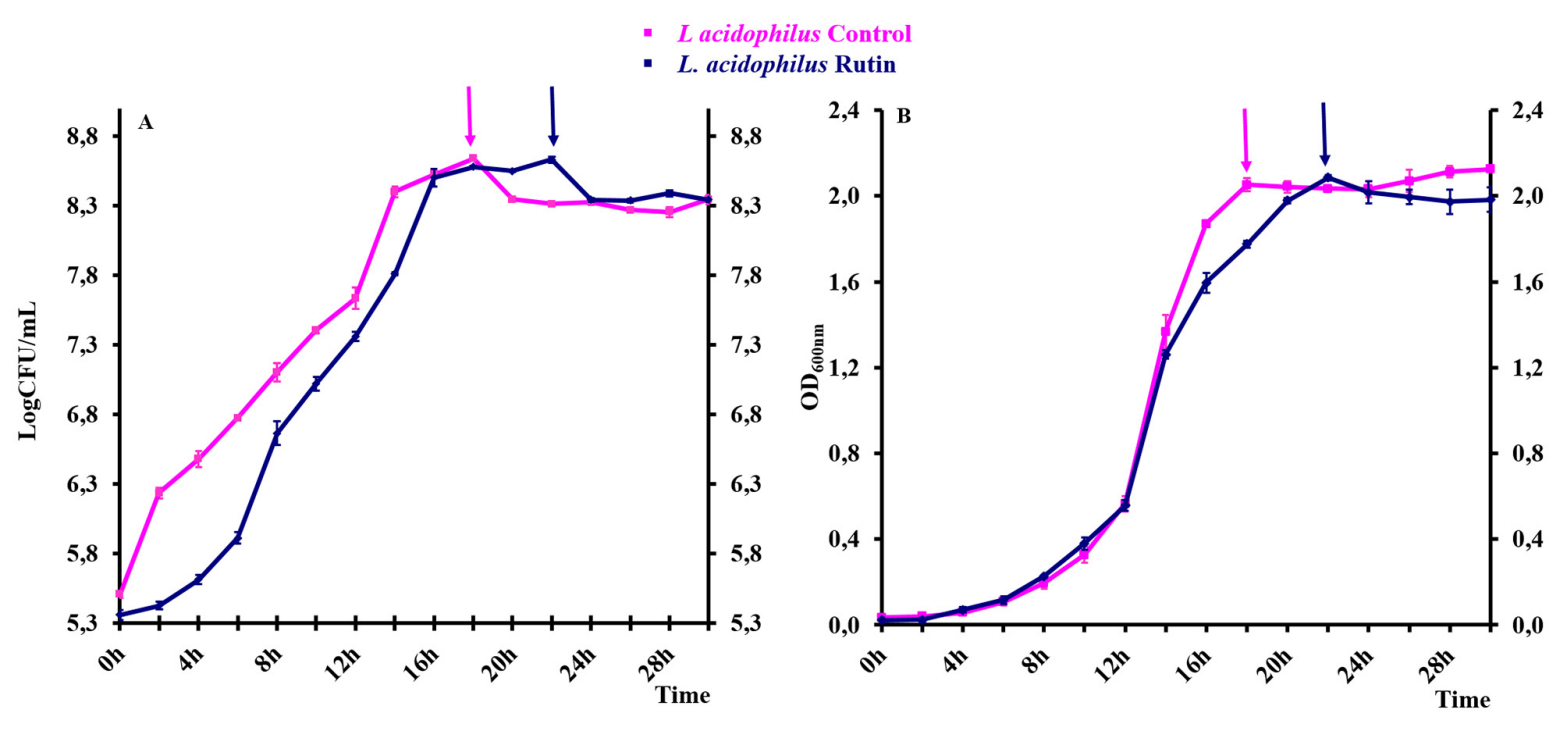
Growth curves of *L*. *acidophilus* in control conditions and in the presence of rutin. (A) Growth curves of *L*. *acidophilus* obtained by plate counting. (B) Growth curves of *L*. *acidophilus* obtained measuring the optical density at 600 nm. Arrows indicate the times of bacterial cell collection for the further proteomic analyses. Values are the means of four independent experiments.

*L*. *acidophilus* DSMZ 20079 (Leibniz Institute DSMZ-German Collection of Microorganisms and Cell Cultures, Germany) samples were prepared by using a two-step procedure. Bacteria from slants kept at 4°C were inoculated into DE MAN, ROGOSA, SHARPE broth (MRS, Thermo Scientific, Basingstoke, UK) and incubated for 24 h at 37°C. Then, the bacterial suspension (1%, 2.0×10^8^ colony forming unit/mL (CFU/mL)) was used to inoculate fresh medium without (control sample) and with rutin (250 μg/mL final concentration). As reported by Duda-Chodak, this rutin concentration was unable to exert any antimicrobial activity on several bacteria species including lactobacilli [26]. For each condition, four bacterial growths were monitored by plate counting on MRS agar incubated at 37°C for 48 h and measuring the optical density at 600 nm (OD_600_) every two hours, in order to obtain the growth curves (S1 Table, Fig 1). Bacterial cells from two biological replicates for each condition were collected by centrifugation (7700 g for 15 min at 4°C) at early stationary phase defined as time 18 h for control sample (OD_600_ = 2.05, CFU = 4.37×10^8^) and time 22 h for *L*. *acidophilus* grown in the presence of rutin (OD_600_ = 2.09, CFU = 4.28×10^8^) and used to perform the proteomic experiments (S1 Table). Cell pellets from 50 mL of each bacterial culture were washed twice with PBS and submitted to enzymatic lysis with mutanolysin (134 units for 1.8×10^10^ cells, Sigma-Aldrich, St. Louise, MO, USA) in 20 mM Tris-HCl, 10 mM MgCl_2_, 0.5 M sucrose pH 7.4 containing a cocktail of protease inhibitors (1/100 v/v, Sigma-Aldrich) for 2 h at 37°C. DNase I and Rnase A reactions (enzyme to substrate ratio of 1:100 v/v, Sigma-Aldrich) were carried out for 30 min at 37°C. Finally, protein precipitation was performed using 10 volumes of acidic acetone (1 mM HCl final concentration)/methanol (50/50 v/v) at −20°C for 18 h. Protein pellets were finally dissolved in buffer solution (8 M urea, 4% (w/v) CHAPS, 40 mM Tris–HCl, 1% DTT) and protein concentration was determined using the Bradford assay [27].

### 2-DE and image analysis

Protein samples were separated by 2-DE [28,29]. Samples containing 250 μg proteins were diluted in IPG strip rehydration buffer (8 M urea, 2% (w/v) CHAPS, 0.5% IPG buffer, 2% DTT and 0.5% bromophenol blue) and loaded on 24-cm IPG strips pH 4–7 (GE Healthcare, Amersham Biosciences AB, Uppsala, Sweden).

Isoelectric focusing (IEF) was carried out at 20°C on the Ettan IPGphor Isoelectric Focusing System (GE Healthcare) to 69.5 kVh totally. After focusing, the IPG strips were equilibrated for 15 min in the equilibration buffer (50 mM Tris-HCl, pH 8.8, 6 M urea, 30% glycerol, 2% SDS, 0.5% bromophenol blue) containing 1% DTT, and for further 15 min in the same equilibration buffer containing 2.5% iodoacetamide. The second dimension gel electrophoresis (SDS-PAGE) was carried out using the vertical slab separation unit Ettan Dalt II System (GE Healthcare). Homogeneous SDS 12.5% polyacrylamide gel was used in a Laemmli system at a constant current of 15 mA/gel and at 10°C [30].

After separation, 2-DE gels were stained using Coomassie Blue Colloidal dye (Sigma-Aldrich).

Stained gels were scanned with an Image Scanner (GE Healthcare) at 300 dpi resolution and analyzed with Image-Master 2D Platinum v.6 software (GE Healthcare) as already reported [31]. Briefly, spot detection was carried out using the optimized setting values for spot intensity, spot area and saliency, determined by applying real-time filters in order to minimize the detection of artifacts. After spot detection, manual spot editing was carried out to remove artifacts that escaped the filtering process. Three gels from the two biological replicates for each growth condition were used to create the two match sets. Relative spot volume (% vol), i.e. digitized staining intensity integrated over the area of the individual spot divided by the sum of volume of all spots in the gel and multiplied by 100, was used for spot quantification [32]. Spots present in all the gels of each group and exhibiting a significant intensity difference (> 1.5 fold change) between the two samples with a P value < 0.05, using the two tailored Student’s t-test for equal or unequal variance (depending on the calculated variance of spots), were considered to contain differentially expressed proteins.

### Protein identification and functional classification

Spots were excised from 2-DE gels and in-gel triptyc digestion was carried out following the procedure described by Shevchenko *et al*. [33]. MALDI-TOF-MS analyses were carried out on a Voyager DE PRO mass spectrometer (Applied Biosystems, Foster City, CA, USA) operating in positive-ion reflectron mode. Mass spectra were calibrated using as internal standards the monoisotopic peaks of angiotensin (m/z 931.5154) and adrenocorticotropic hormone (ACTH) fragment 18–39 (m/z 2465.1989) and data were processed using the DataExplorer 5.1 software (Applied Biosystems). Protein identification was achieved based on the mass spectral data using the Mascot Wizard tool for searches against the NCBInr database (http://www.matrixscience.com/). Parameters for all searches were as follows: all entries as taxonomic category, trypsin as enzyme, carbamidomethyl as fixed modification for cysteine residues, oxidation as variable modification for methionine residues, up to one missed cleavage and up to 50 ppm as mass tolerance.

Identified proteins were classified on the basis of their biological functions using the bioinformatics resource KEGG (Kyoto Encyclopedia of Genes and Genomes, http://www.genome.jp/kegg/).

Integrated function and protein interactions were explored using the database and web resource STRING v.9.1 (Search Tool for the Retrieval of Interacting Genes/Proteins, http://string-db.org/). Active prediction methods used in our analysis were neighbourhood, coexpression, experiments, co-occurrence, databases and text mining, using custom confidence value of 0.600 [34].

## Results

### Influence of rutin on bacterial growth

The impact of rutin on *L*. *acidophilus* growth was clearly shown by the growth curves reported in Fig 1. The polyphenol had no inhibitory effect, but it slightly affected bacterial growth, with *L*. *acidophilus* cells reaching the early stationary phase with a delay of four hours in the presence of rutin (22 h) comparing to control cells (18 h). However, no substantial differences were observed in cell viability in the stationary phase (S1 Table). This observation suggested that a phase of adaptation to rutin was necessary to *L*. *acidophilus*, before it could initiate cell multiplication. Based on these results, the proteomic study was carried out on bacterial cells grown for 22 h in the presence of rutin and for 18 h in the control medium.

### Proteomic analyses and functional classification of identified proteins

**Fig 2:**
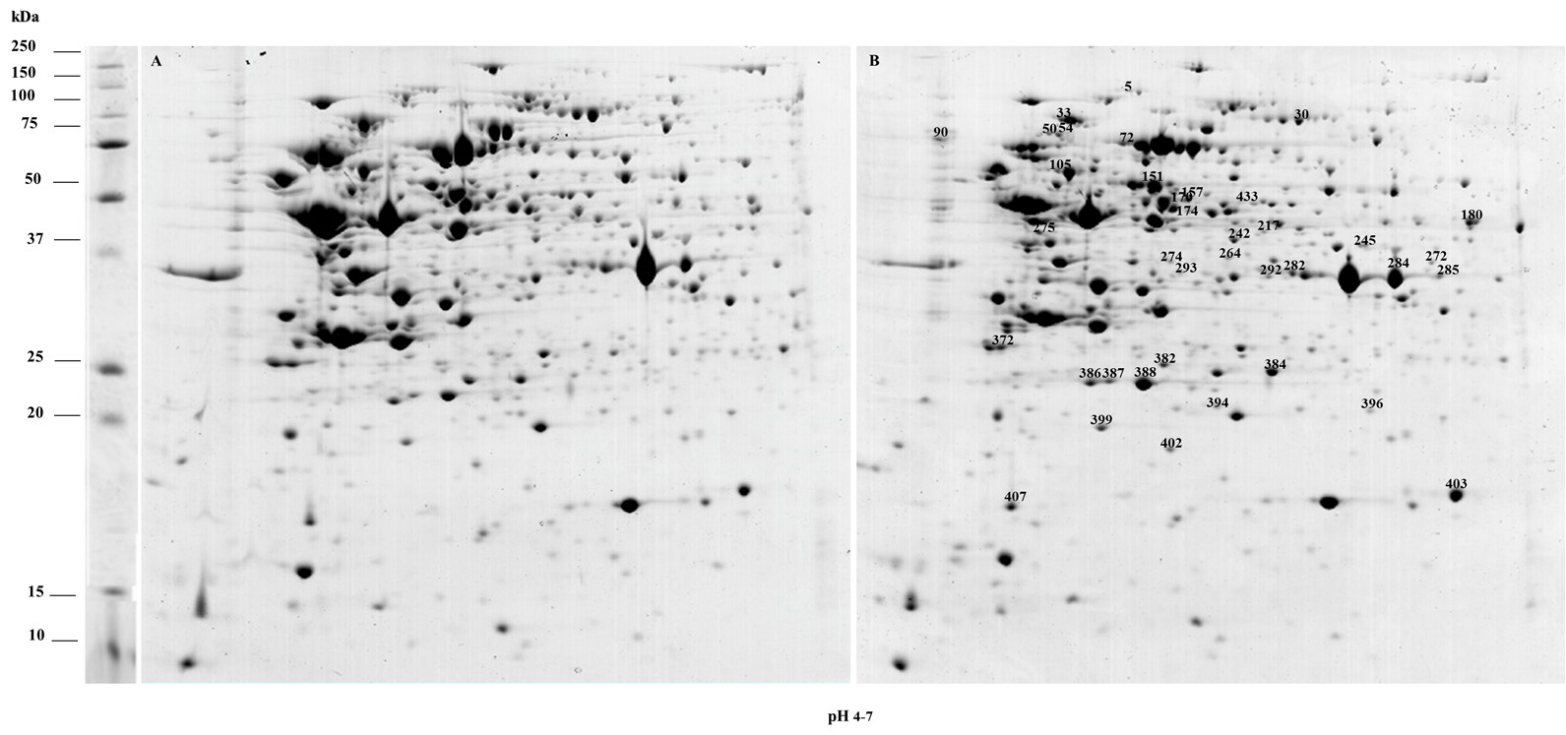
Representative 2-DE gels of *L*. *acidophilus* proteome. (A) 2-DE gel obtained from the proteome of *L*. *acidophilus* grown under control conditions. (B) 2-DE gel obtained from the proteome of *L*. *acidophilus* grown in the presence of rutin. Spots exhibiting significant differences in mean intensities are indicated.

2-DE maps obtained from the analysis of *L*. *acidophilus* proteome contained about 430 spots (Fig 2). By comparing the 2-DE map of *L*. *acidophilus* grown in the presence of rutin to that of control sample, 38 spots displayed a mean intensity variation higher than a 1.5 factor and 35 proteins contained in these spots were identified (Table 1).

**Table 1:** Identification of differentially expressed proteins.

Spot	Accession Number (NCBInr)	Protein	Organism	MW	pI	Score	Matched Peptides	Coverage
5	gi|58337125	isoleucyl-tRNA synthetase	*L*. *acidophilus* NCFM	106576	5.12	207	20/30	29
30	gi|58336392	ribonucleoside triphosphate reductase	*L*. *acidophilus* NCFM	83982	5.62	491	47/76	63
33	gi|58336628	elongation factor G	*L*. *acidophilus* NCFM	76806	4.94	221	17/30	40
50	gi|58336963	ATP-dependent Clp protease, ATP-binding subunit ClpE	*L*. *acidophilus* NCFM	81748	5.00	221	23/32	39
54	gi|58336963	ATP-dependent Clp protease, ATP-binding subunit ClpE	*L*. *acidophilus* NCFM	81748	5.00	283	35/69	51
72	gi|58337255	pyruvate kinase	*L*. *acidophilus* NCFM	63136	5.23	227	27/59	58
90	gi|58337121	cell division protein FtsZ	*L*. *acidophilus* NCFM	48119	4.55	135	15/60	42
105	gi|58336743	molecular chaperone GroEL	*L*. *acidophilus* NCFM	57785	4.98	269	32/65	52
151	gi|58337766	glutamine synthetase	*L*. *acidophilus* NCFM	50672	5.17	227	27/71	48
157	gi|58337885	seryl-tRNA synthetase	*L*. *acidophilus* NCFM	49813	5.31	310	36/65	65
170	gi|58337265	30S ribosomal protein S1	*L*. *acidophilus* NCFM	44414	5.15	220	16/30	55
174	gi|58337282	ATP-dependent protease ATP-binding subunit HslU	*L*. *acidophilus* NCFM	52775	5.26	367	38/64	55
180	gi|58337501	pyridine mercuric reductase	*L*. *acidophilus* NCFM	49135	6.08	361	42/67	63
217	gi|58337130	tRNA-specific 2-thiouridylase MnmA	*L*. *acidophilus* NCFM	42426	5.50	231	20/67	59
242	gi|58337163	aspartate aminotransferase	*L*. *acidophilus* NCFM	43142	5.44	327	24/46	62
245	gi|58337903	multiple sugar ABC transporter ATPase	*L*. *acidophilus* NCFM	40533	5.87	264	22/44	56
264	gi|58337019	glyceraldehyde-3-phosphate dehydrogenase	*L*. *acidophilus* NCFM	36643	5.92	128	14/68	47
272	gi|489643333	outer surface protein	*L*. *acidophilus* NCFM	41656	6.08	202	17/37	45
274	gi|58336976	isomerase	*L*. *acidophilus* NCFM	38113	5.22	238	14/23	55
275	gi|58336405	D-lactate dehydrogenase	*L*. *acidophilus* NCFM	39177	4.96	215	28/63	76
282	gi|58337217	citrate lyase ligase	*L*. *acidophilus* NCFM	39455	5.57	246	28/74	62
284	gi|58337019	glyceraldehyde-3-phosphate dehydrogenase	*L*. *acidophilus* NCFM	36643	5.92	344	28/67	93
285	gi|58337019	glyceraldehyde-3-phosphate dehydrogenase	*L*. *acidophilus* NCFM	36643	5.92	341	30/52	76
292	gi|58336524	oxidoreductase	*L*. *acidophilus* NCFM	36225	5.57	241	22/29	64
293	gi|58337614	branched-chain amino acid aminotransferase	*L*. *acidophilus* NCFM	37926	5.31	222	22/50	53
372	gi|58337021	triosephosphate isomerase	*L*. *acidophilus* NCFM	27770	4.72	259	24/81	72
382	gi|58337926	elongation factor Pphosphoglycerate mutase	*L*. *acidophilus* NCFM	21060	5.26	129	17/42	59
	gi|58337936		*L*. *acidophilus* NCFM	25036	5.17	168	14/42	74
384	gi|58337337	glutamine ABC transporter ATP-binding protein	*L*. *acidophilus* NCFM	28347	5.58	246	32/45	81
386	gi|58337608	elongation factor P	*L*. *acidophilus* NCFM	20849	5.07	72	6/14	29
387	gi|58338068	two-component response regulator	*L*. *acidophilus* NCFM	25504	5.12	99	7/21	30
388	gi|499573661	NAD-dependent dehydratase	*L*. *acidophilus*	23620	5.35	297	22/31	70
394	gi|489643392	ribosome recycling factor	*L*. *acidophilus*	20675	5.66	194	16/33	42
396	gi|58337527	heat shock protein GrpE	*L*. *acidophilus* NCFM	22044	5.79	163	18/33	63
399	gi|58337015	ATP-dependent Clp protease proteolytic subunit ClpP	*L*. *acidophilus* NCFM	21400	5.26	143	14/46	61
402	gi|58337281	ATP-dependent protease peptidase subunit HslV	*L*. *acidophilus* NCFM	18714	5.21	176	16/35	57
403	gi|58337284	hypothetical protein LBA0987	*L*. *acidophilus* NCFM	15561	6.07	190	16/50	70
407	gi|58337297	trp repressor binding protein	*L*. *acidophilus* NCFM	16992	4.81	145	13/52	72
433	gi|58336560	bifunctional N-acetylglucosamine-1-phosphate uridyltransferase/glucosamine-1-phosphate acetyltransferase	*L*. *acidophilus* NCFM	50162	5.57	140	20/70	41

The analysis of adjacent spots in the 2-DE maps led to the identification of the same protein in two cases, i.e. glyceraldehyde-3-phosphate dehydrogenase was identified in spots 264, 284 and 285, and ATP-dependent Clp protease subunit ClpE was identified in spots 50 and 54, thus indicating the presence of isoforms probably due to post-translational modifications. Two co-migrating proteins were identified in spot 382; this spot was not taken into account in the further analyses. Twenty-six proteins were present in higher abundance and eight proteins were present in lower abundance in the 2-DE map of cells grown in the presence of rutin. Spot 272, containing an outer surface protein, was detectable only in the 2-DE map of *L*. *acidophilus* grown in the presence of rutin.

**Fig 3:**
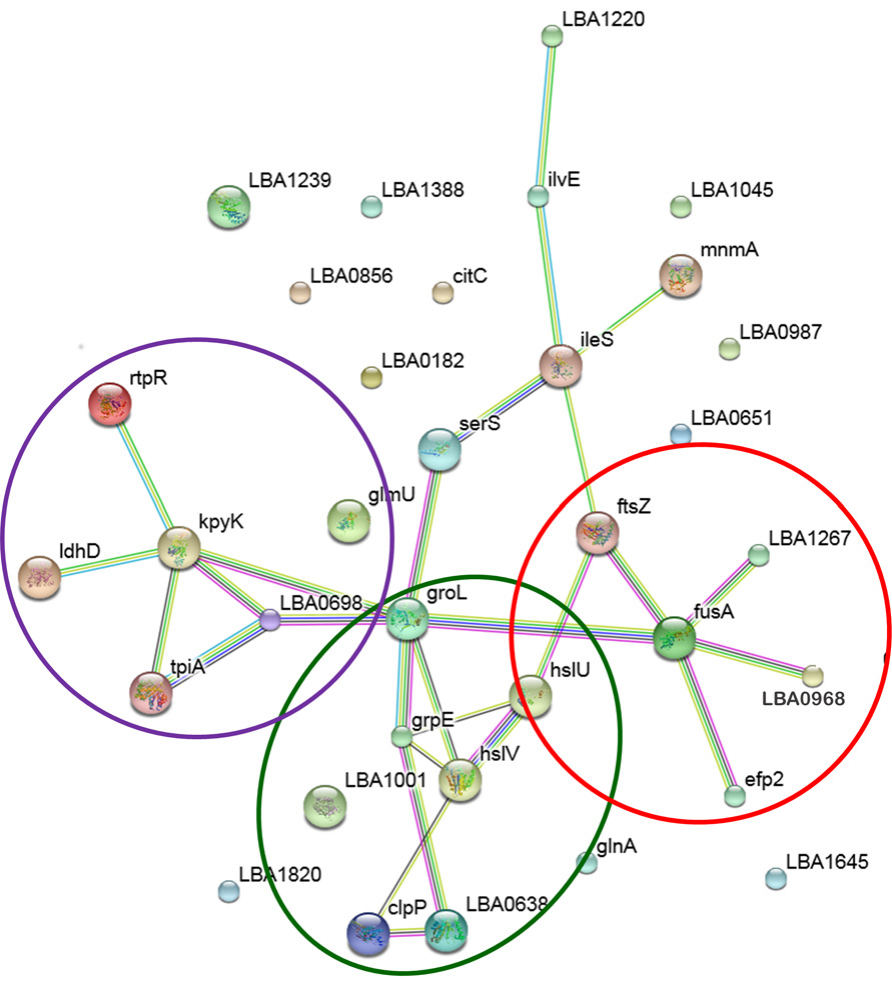
Interaction network (as displayed by EMBL STRING) of the identified proteins. Green lines indicate neighborhood, blue lines indicate co-occurrence, black lines indicate co-expression, purple lines indicate experiments, light blue lines indicate databases and yellow lines indicate text-mining. The network was obtained with a confidence score of 0.600. Main functional modules are circled.

Functional classification of identified proteins was performed by KEGG and protein‑protein interactions were analyzed using the web resource STRING (Table 2, Fig 3). The network is presented under evidence view, whereby different line colors represent the types of evidence for the association, whereas proteins are represented as nodes. Fig 3 shows the interaction between 22 out of 34 identified proteins, found to be linked either directly or indirectly through one or more interacting proteins, suggesting the existence of functional linkages. Notably, STRING analysis highlighted three main functional modules forming tightly connected clusters. The first functional module (circled in purple in Fig 3) included proteins involved in the energy metabolism (*kpyK*, *tpiA*, *ldhD*, LAB0698, Table 2), the second one (circled in green in Fig 3) included proteins responsible for protein folding and degradation (*groEL*, *grpE*, LBA0638, *clpP*, *hslIV*, *hslIU*, Table 2), the last one (circled in red in Fig 3) included ribosomal proteins and translation factors (*fusA*, *efp2*, LBA0968, LBA1267). These functional analyses highlighted that rutin induced in *L*. *acidophilus* metabolic changes mainly affecting carbohydrate and amino acid metabolism. Moreover, protein synthesis and molecular processes necessary to assure proper protein folding and avoid protein aggregation were activated by rutin.

**Table 2:** Functional classification of the identified proteins and regulation of their abundance.

Protein	Gene	Locus tag	Functional classification	Regulation ^a^
**CARBOHYDRATE METABOLISM**				
glyceraldehyde-3-p dehydrogenase		LBA0698	Glycolysis / Gluconeogenesis	UP^b^(2.5)
triosephosphate isomerase	*tpiA*	LBA0700	Glycolysis / Gluconeogenesis / Fructose and mannose metabolism	DOWN(-1.8)
pyruvate kinase	*kpyK*	LBA0957	Glycolysis / Gluconeogenesis / Pyruvate metabolism	UP(1.8)
D-lactate dehydrogenase	*ldhD*	LBA0055	Pyruvate metabolism	DOWN(-1.7)
isomerase		LBA0651	Pentose phosphate pathway	UP(1.6)
**AMINO ACIDS BIOSYNTESIS AND METABOLISM**				
aspartate aminotransferase		LBA0856	Lysine biosynthesis	UP(1.6)
branched-chain amino acid aminotransferase	*ilvE*	LBA1341	Valine, leucine and isoleucine biosynthesis or degradation	UP(1.6)
glutamine synthetase	*glnA*	LBA1501	Alanine, aspartate and glutamate metabolism / Arginine and proline metabolism	DOWN(-1.6)
**NUCLEOTIDE METABOLISM**				
ribonucleoside triphosphate reductase	*rtpR*	LBA0041	Purine metabolism	DOWN(-1.8)
**AMINO SUGAR AND NUCLEOTIDE SUGAR METABOLISM**				
bifunctional N-acetylglucosamine-1-phosphate uridyltransferase/glucosamine-1-phosphate acetyltransferase	*glmU*	LBA0219		DOWN(-1.8)
**TRANSPORT SYSTEM**				
glutamine ABC transporter ATP-binding protein		LBA1045		UP(7.8)
multiple sugar-binding ABC-transporter ATPase		LBA1645		DOWN(-2.0)
**TRANSLATION**				
isoleucyl-tRNA synthetase	*ileS*	LBA0817		DOWN(-1.8)
seryl-tRNA synthetase	*serS*	LBA1626		DOWN(-1.8)
ribosome recycling factor Rrf		LBA1267		UP(2.8)
30S ribosomal protein S1	*rpsA*	LBA0968		UP(2.9)
elongation factor G	*fusA*	LBA0289		UP(1.6)
elongation factor P	*efp2*	LBA1335		UP(1.6)
**TRANSCRIPTION**				
two-component response regulator		LBA1820		UP(2.0)
**SIGNAL TRANSDUCTION**				
citrate lyase ligase	*citC*	LBA0914	Two-component system	UP(1.6)
**FOLDING, SORTING AND DEGRADATION**				
ATP-dependent Clp protease, ATP-binding subunit ClpE	*clpE*	LBA0638		UP^b^(5.3)
ATP-dependent Clp protease proteolytic subunit	*clpP*	LBA0694		UP(1.6)
ATP-dependent protease ATP-binding subunit HslU	*hslU*	LBA0985		UP(1.7)
ATP-dependent protease subunit HslV	*hslV*	LBA0984		UP(1.6)
heat shock protein GrpE	*grpE*	LBA1248		UP(1.6)
molecular chaperone GroEL	*groEL*	LBA0406		UP(1.6)
tRNA-specific 2-thiouridylase MnmA	*mnmA*	LBA0822	Sulfur relay system	UP(1.7)
**OXIDATION-REDUCTION PROCESS**				
oxidoreductase		LBA0182		UP(1.6)
trp repressor binding protein		LBA1001		UP(1.7)
pyridine mercuric reductase		LBA1220		UP(2.3)
**MISCELLANEOUS**				
cell division protein FtsZ	*ftsZ*	LBA0812		UP(1.6)
NAD-dependent dehydratase				UP(2.2)
hypothetical protein LBA0987		LBA0987		UP(2.1)
outer surface protein		LBA1239		spot detected only in 2-DE map of *L*. *acidophilus* grown with rutin

^a^Changes in protein levels are reported as the ratio between the relative spot volume from *L*. *acidophilus* grown in the presence of rutin and *L*. *acidophilus* grown in control conditions (Vrutin/Vcontrol) for increased proteins and as the negative reciprocal values (-Vcontrol/Vrutin) for decreased proteins. Changes in protein levels >1.5 have been considered significant.

^b^Average fold change for proteins contained in more than one spot has been calculated considering the relative spot volume of all the spots containing the same protein and a fold change >1.5 has been considered significant.

## Discussion

Rutin is a polyphenol relevant for its antioxidant properties and its aglycone form (quercetin) has well documented positive effects on human health [4]. As most polyphenols, rutin is not absorbed in the small intestine and is metabolized in the gut by indigenous microbiota. Therefore, bioavailability and biological activity of rutin depend on the potential interaction with intestinal bacteria [7–9]. However, information on the molecular mechanisms underlying this interaction are still scarce. Lactobacilli could be leading actors in polyphenol degradation as they are able to efficiently hydrolyze the glycosidic moiety of flavonols and flavanones [3].

In this context, a differential proteomic study was performed to investigate the adaptive response of a potentially probiotic *L*. *acidophilus* strain to the presence of rutin in the growth medium. In our conditions, the presence of rutin induced a growth retardation, although cell viability in the stationary phase did not changed significantly (Fig 1). A similar effect was also displayed by other phenolic compounds on some *Lactobacillus* species, in particular tannic acid on *L*. *plantarum* [13], and p-coumaric acid on *L*. *casei* [15]. On the contrary, the polyphenol extract from *Sesbania grandiflora* flower, containing rutin as a major flavonoid, had a growth promoting property on *L*. *acidophilus*. However, this effect could be partially due to the synergic action of other polyphenols present in the extract [25].

Proteomic data were analyzed by STRING, which is a web resource aimed to predict protein-protein interactions, considering both physical and functional associations and integrating information from numerous sources, including experimental repositories, computational prediction methods and public text collections [34]. STRING analysis led to define three main functional modules forming tightly connected clusters that included proteins involved in the energy metabolism, protein folding and degradation, and ribosomal proteins and translation factors (Fig 3).

In particular, rutin induced stress conditions in bacterial cells, that elicited a response by activating the production of proteins belonging to the protein quality control systems such as the folding and degradation machineries to promote refolding and proper protein assembly, prevent aggregation phenomena and hydrolyze damaged proteins. These molecular mechanisms are regarded as general stress response systems in lactobacilli [35]. In fact, an increased abundance of heat shock protein GrpE, molecular chaperone GroEL, and ATP-binding subunits ClpE and ClpP of ATP-dependent Clp protease (included in the cluster circled in green in Fig 3) was observed in cells grown in the presence of rutin. Moreover, the overexpression of HslV protease and HslU ATPase that compose the two-component proteasome-related prokaryotic system (Table 2), the bacterial homologue of the eukaryotic 20S proteasome core particle, well documented the need to degrade damaged or misfolded proteins in order to maintain cellular homeostasis [36].

In addition, rutin induced the activation of glycolysis as the key enzymes glyceraldehyde-3-phosphate dehydrogenase and pyruvate kinase (included in the cluster circled in purple in Fig 3) were overexpressed in cells grown in the presence of this molecule. An increase of ATP production should be necessary to sustain the activity of the ATP-dependent folding and degradation systems. A similar trend has been also associated to the effect of tannic acid on *L*. *plantarum* [13].

The increased level of pyruvate kinase could also parallel with the higher expression level of branched-chain amino acid aminotransferase. This enzyme is involved in both biosynthesis and catabolism of branched-chain amino acids (BCAA) and catalyzes the final step of the conversion of pyruvate into BCAA in the biosynthetic pathway and the first step of BCAA degradation that leads to the production of α-keto acids in the catabolitic pathway. These are precursors of branched chain fatty acids, molecules that affect bacterial membrane fluidity. In *Streptococcus mutans*, BCAA aminotransferase played a key role in conferring acid tolerance and the biosynthesis of BCAA was proposed as a way to maintain proper intracellular pH conditions [37,38]. Overexpression of this protein was also reported in acid-stressed *L*. *plantarum* 423 cells [39]. In this light, it could be suggested that this enzyme might also play a role in the adaptive response to rutin in *L*. *acidophilus*.

Our results also suggested that the presence of rutin could negatively affect cell wall integrity. In fact, a decreased amount of bifunctional N-acetylglucosamine-1-phosphate uridyltransferase/glucosamine-1-phosphate acetyltransferase (GlmU) and D-lactate dehydrogenase in bacteria grown in the presence of rutin was found. GlmU is a bi-functional enzyme that catalyzes the last steps of the synthesis of UDP-N-acetylglucosamine (UDP-GlcNAc), one of the main precursors of bacterial cell wall peptidoglycan, from fructose-6-phosphate [40,41] while D-lactate dehydrogenase catalyzes the conversion of pyruvate into D-lactic acid, the last residue incorporated in the muramoyl-pentadepsipeptide peptidoglycan precursor [42].

We observed an increased amount of proteins with oxidoreductase activity in cells grown with rutin that could trigger their expression to withstand oxidative stress, as already reported for other phenolic compounds [14]. However, the overexpression of oxidative stress related proteins could also take part in the general stress response mechanism.

Our data indicated that the presence of rutin strongly affected protein synthesis and cells needed to promote ribosomal activity. Worth noting, the abundance of 30S ribosomal protein S1, EF-P, EF-G and ribosome recycling factor Rrf, that are connected in the cluster circled in red in Fig 3, increased in *L*. *acidophilus* grown in the presence of rutin. EF-G catalyzes the movement of the tRNA-mRNA complex within the ribosome during translocation, while ribosome recycling factor Rrf, in conjunction with EF-G and Initiation Factor 3 (IF3), promotes the dissociation of the ribosome into its subunits in order to recycle ribosomes for a new round of translation. EF-P stimulates the first step of peptide bond formation through an interaction with the ribosome and initiator tRNA and rescues the ribosome stalling that occurs mainly during the synthesis of proteins containing consecutive prolines [43,44]. Recently, it has also been indicated a key role of EF-P in the translational regulation of a subset of proteins involved in stress tolerance [43,45]. Proteomic studies already showed that the growth in the presence of tannic acid induced an overexpression of ribosomal proteins and elongation factors in *L*. *plantarum* also associated to the prolongation of cell viability [13]. Moreover, the abundance of these proteins mostly increased in response to acid and bile exposure in other lactobacilli and bifidobacteria [46].

Interestingly we observed that cells grown in the presence of rutin expressed an outer surface protein that contained a glycoside hydrolase domain. This protein showed a high sequence identity with a PTS (PhosphoTransferase System) associated protein of other lactobacilli, a complex system involved in the uptake and metabolism of oligosaccharides [47]. As a biological function of this protein has not been established yet, it could not be ruled out a specific role in the degradation of the glycosidic moiety of conjugated polyphenols.

In conclusion, this study provided a first overview of the lactobacilli-flavonoids interplay and showed that adaptive response to rutin modulated the expression level of proteins known to be involved in general stress response mechanisms, in particular to acid and oxidative stresses. Due to cross-protection phenomenon [35,48], drastic stress conditions faced by lactobacilli during the gastro-intestinal transit should prepare these bacteria to better tolerate diet polyphenols so that they might contribute to the metabolism of these compounds.

## Supporting Information

S1 Table *L*. *acidophilus* growths in control conditions and in the presence of rutin. Four bacterial growth for each condition were monitored by optical density at 600 nm (OD sheet) and by plate counting (CFU sheet).
(XLSX)
